# *Methylenetetrahydrofolate reductase* C677T polymorphism is not associated with the risk of nonsyndromic cleft lip/palate: An updated meta-analysis

**DOI:** 10.1038/s41598-020-58357-0

**Published:** 2020-01-30

**Authors:** Mohammad Moslem Imani, Negin Golchin, Mohsen Safaei, Farzad Rezaei, Hooshyar Abbasi, Masoud Sadeghi, Pia Lopez-Jornet, Hamid Reza Mozaffari, Roohollah Sharifi

**Affiliations:** 1 Department of Orthodontics, School of Dentistry, Kermanshah University of Medical Sciences, Kermanshah, 6713954658 Iran; 2 Students Research Committee, Kermanshah University of Medical Sciences, Kermanshah, 6715847141 Iran; 3 Advanced Dental Sciences Research Laboratory, School of Dentistry, Kermanshah University of Medical Sciences, Kermanshah, 6713954658 Iran; 4 Department of Oral and Maxillofacial Surgery, Kermanshah University of Medical Sciences, Kermanshah, 6713954658 Iran; 5 Medical Biology Research Center, Kermanshah University of Medical Sciences, Kermanshah, 6714415185 Iran; 6 Facultad de Medicina y Odontologia Universidad de Murcia, Hospital Morales Meseguer, Clinica Odontologic Adv Marques Velez s/n, 30008 Murcia, Spain; 7 Department of Oral and Maxillofacial Medicine, School of Dentistry, Kermanshah University of Medical Sciences, Kermanshah, 6713954658 Iran; 8 Department of Endodontics, School of Dentistry, Kermanshah University of Medical Sciences, Kermanshah, 6713954658 Iran

**Keywords:** Genetics, Molecular medicine

## Abstract

Both genetic and environmental factors affect the risk of orofacial clefts. The present meta-analysis aimed to evaluate the association between *methylenetetrahydrofolate reductase (MTHFR)* C677T polymorphism and risk of nonsyndromic cleft lip/palate (NSCL/P) in cases-control studies. The PubMed/Medline, Scopus, Web of Science, and Cochrane Library databases were searched up to April 2019 with no restrictions. The odds ratios (ORs) and 95% confidence intervals (CIs) in all analyses were calculated by Review Manager 5.3 software. The funnel plot analysis was carried out by the Comprehensive Meta-Analysis version 2.0 software. Subgroup analysis, meta-regression, and sensitivity analysis were performed for the pooled analyses. Thirty-one studies reviewed in this meta-analysis included 4710 NSCL/P patients and 7271 controls. There was no significant association between *MTHFR* C677T polymorphism and NSCL/P susceptibility related to allelic model (OR = 1.04; P = 0.49), homozygote model (OR = 1.11; P = 0.35), heterozygote model (OR = 0.99; P = 0.91), dominant model (OR = 1.00; P = 0.96), or recessive model (OR = 1.08; P = 0.23). There was no significant association between *MTHFR* C677T polymorphism and NSCL/P susceptibility based on the ethnicity or the source of cases. There was a significant linear relationship between the year of publication and log ORs for the allele model. The results of the present meta-analysis failed to show an association between *MTHFR* C677T polymorphism and NSCL/P susceptibility. The subgroup analyses based on the ethnicity and the source of cases further confirmed this result.

## Introduction

Non-syndromic cleft lip/palate (NSCL/P) is a common birth defect worldwide^1^. In low- and middle-income countries, around 1/730 children is born with cleft lip/palate^2^. A multifactorial model of genetic inheritance has been recommended for NSCL/P based on the interaction of genetic and environmental factors^1^. Several lines of evidence have proven a significant association between polymorphism of genes connected to folate metabolism and increased risk of orofacial clefts^3^. Among genes related to folate metabolism, 5,10-*methylenetetrahydrofolate reductase* (*MTHFR*) reportedly has the highest association with NSCL/P. This gene is located on chromosome 1 at 1p36.3 and translates to MTHFR enzyme that catalyzes the conversion of 5,10-methylenetetrahydrofolate to 5-methyltetrahydrofolate, a cosubstrate for homocysteine remethylation to methionine^4,5^. MTHFR is a fundamental enzyme in folate metabolism and DNA synthesis, and *MTHFR* rs1801133 (C677T) is one of the most common polymorphisms which diminishes the enzyme activity^6–8^. Regulation of MTHFR activity is pivotal to maintain optimal cellular levels of methionine and S-adenosylmethionine^5^. Folate supplementation or its adequate dietary intake during pregnancy has been shown to prevent or decrease NSCL/P susceptibility^9^. Nutritional factors, such as the adequacy of folic acid in the mother’s diet, are clearly important, but other potential disturbances in ovulation or development of fetus may be due to the activity of key factors such as the MTHFR enzyme in folate metabolism^10,11^. In addition, the role of other polymorphisms of folate metabolism has been proven in recent meta-analyses^7,12,13^. There are six published meta-analyses related to our topic in the literature^7,8,14–17^. However, several other original articles have been recently published. Thus, we aimed to evaluate the association between *MTHFR* C677T and risk of NSCL/P in an updated meta-analysis of cases-control studies.

## Materials and Methods

### Protocol

The Preferred Reporting Items for Systematic Reviews and Meta-Analyses (PRISMA) guideline was applied for designing this meta-analysis^18^.

### Search strategy

One author (N.G) accomplished the initial search and another author (M.S) re-checked the retrieved articles; disagreements between the two authors were resolved by a third author (M.M.I). A comprehensive search was conducted on the association between *MTHFR* rs1801133 C > T (C677T) polymorphism and NSCL/P susceptibility in PubMed/Medline, Scopus, Web of Science, and Cochrane Library databases up to April 2019. The search keywords were: (“cleft lip” or “cleft palate” or “orofacial cleft” or “oral cleft”) and (“methylenetetrahydrofolate reductase” or “MTHFR”). The databases were searched without any restrictions. Manual search of all references quoted in published meta-analyses/reviews related to the topic was done by another author (M.S).

### Eligibility criteria

Inclusion criteria: (a) original studies; (b) studies reporting the relationship between *MTHFR* C677T polymorphism and the NSCL/P susceptibility; (c) studies designed as case-control studies; (d) studies providing sufficient data about the alleles and genotypes of *MTHFR* C677T polymorphism in case and control groups. Exclusion criteria: (a) studies not related to the relationship between *MTHFR* C677T polymorphism and the NSCL/P susceptibility; (b) duplicate studies/erratum; (c) review/meta-analysis, letter to editors, commentaries, and conference papers; (d) family-based studies; (e) case-parent triads studies; (f) studies inconsistent with the Hardy-Weinberg equilibrium (HWE) about the control group; (g) studies containing overlapping data.

### Data extraction

Two authors (N.G and M.M.I) independently retrieved the data from each study included in this systematic review based on the eligibility criteria. Disagreements between the two authors were resolved through further discussion. The extracted data are presented in Tables 1 and 2.

**Table 1:** Characteristics of the studies included in this meta-analysis (n = 31).

First author, publication year	Country	Ethnicity	No. of cases/controls	Source of case	Genotype method
Shaw, 1998^27^	USA	Mixed	310/383	PB	RFLP-PCR
Gaspar, 1999^29^	Brazil	Mixed	77/113	HB	PCR
Martinelli, 2001^28^	Italy	Caucasian	116/106	PB	RFLP-PCR
Grunert, 2002^30^	Germany	Caucasian	66/184	HB	PCR
Shotelersuk, 2003^31^	Thailand	Asian	109/202	PB	RFLP-PCR
van Rooij, 2003^32^	Netherlands	Caucasian	105/128	HB	RFLP-PCR
Pezzetti, 2004^33^	Italy	Caucasian	110/289	HB	RFLP-PCR
Wan, 2006^34^	China	Asian	76/60	HB	RFLP-PCR
Brandalize, 2007^35^	Brazil	Mixed	114/100	HB	RFLP-PCR
Chevrier, 2007^36^	France	Caucasian	168/148	HB	RFLP-PCR
Little, 2008^37^	Canada	Mixed	96/224	PB	MS-PCR
Mills, 2008^38^	Ireland	Caucasian	492/1599	HB	RFLP-PCR
Ali, 2009^39^	India	Asian	323/214	PB	RFLP-PCR
Guo, 2009^40^	China	Asian	96/103	HB	PCR
Sozen, 2009^41^	USA	Mixed	179/138	PB	PCR
Mostowska, 2010^42^	Poland	Caucasian	174/176	PB	RFLP-PCR
Chorna, 2011^43^	Ukraine	Caucasian	33/50	HB	RFLP-PCR
Han, 2011^44^	China	Asian	200/213	HB	RFLP-PCR
Semic-Jusufagic, 2012^45^	Turkey	Caucasian	56/76	PB	PCR
Kumari, 2013^46^	India	Asian	467/469	PB	RFLP-PCR
Estandia-Ortega, 2014^47^	Mexico	Mixed	132/370	HB	KASPar assay system
Jahanbin, 2014^48^	Iran	Caucasian	45/101	PB	RFLP-PCR
Murthy, 2014^49^	India	Asian	123/141	HB	RFLP-PCR
Abdollahi-Fakhim, 2015^50^	Iran	Caucasian	65/50	HB	RFLP-PCR
Bezerra, 2015^51^	Brazil	Mixed	140/175	PB	RFLP-PCR
de Aguiar, 2015^52^	Brazil	Mixed	318/598	HB	Real time-PCR
Jiang, 2015^53^	China	Asian	204/226	PB	SEQUENOM MassARRAY
Ramírez-Chau, 2016^54^	Chile	Mixed	165/291	HB	Real time-PCR
Xu, 2016^55^	China	Asian	120/100	PB	PCR
Taslim, 2017^56^	Indonesia	Asian	24/47	HB	RFLP-PCR
Rafik, 2019^57^	Morocco	Mixed	52/182	HB	RFLP-PCR

**Table 2:** Distribution of *MTHFR* C677T polymorphism genotype and allele in NSCL/P patients and controls.

First author, publication year	Case			Control			Case		Control		P-value for HWE in controls
	CC	CT	TT	CC	CT	TT	C	T	C	T	
Shaw, 1998^27^	143	127	40	156	178	49	413	207	790	276	0.87
Gaspar, 1999^29^	30	39	8	49	49	15	99	55	147	79	0.09
Martinelli, 2001^28^	64	22	30	46	43	17	150	82	135	77	0.20
Grunert, 2002^30^	34	26	6	90	69	25	94	38	249	119	0.06
Shotelersuk, 2003^31^	84	25	0	154	46	2	193	25	354	50	0.47
van Rooij, 2003^32^	54	45	6	70	54	4	153	57	194	62	0.09
Pezzetti, 2004^33^	28	58	24	95	151	43	114	106	341	237	0.17
Wan, 2006^34^	13	49	14	31	20	9	75	77	82	38	0.08
Brandalize, 2007^35^	49	46	19	45	41	14	144	84	131	69	0.35
Chevrier, 2007^36^	66	60	22	54	81	33	192	104	189	147	0.17
Little, 2008^37^	39	47	10	94	101	29	125	67	289	159	0.82
Mills, 2008^38^	217	221	54	715	721	163	655	329	2151	1047	0.34
Ali, 2009^39^	225	87	11	176	36	2	537	109	388	40	0.91
Guo, 2009^40^	19	53	24	22	57	24	91	101	101	105	0.27
Sozen, 2009^41^	81	80	18	66	65	7	242	116	197	79	0.07
Mostowska, 2010^42^	81	65	17	78	77	16	227	99	233	109	0.67
Chorna, 2011^43^	12	17	4	22	26	2	41	25	70	30	0.09
Han, 2011^44^	46	106	35	74	110	29	198	176	258	168	0.24
Semic-Jusufagic, 2012^45^	25	28	3	44	24	8	78	34	112	40	0.10
Kumari, 2013^46^	327	126	15	364	100	5	780	156	828	110	0.52
Estandia-Ortega, 2014^47^	38	55	39	55	172	143	131	133	282	458	0.78
Jahanbin, 2014^48^	20	16	7	46	41	14	56	30	133	69	0.32
Murthy, 2014^49^	104	19	0	107	31	3	227	19	245	37	0.67
Abdollahi-Fakhim, 2015^50^	38	25	2	27	22	1	101	29	76	24	0.14
Bezerra, 2015^51^	74	54	12	85	70	20	202	78	240	110	0.34
de Aguiar, 2015^52^	137	145	36	319	231	48	419	217	869	327	0.50
Jiang, 2015^53^	59	107	38	62	108	56	225	183	232	220	0.51
Ramírez-Chau, 2016^54^	44	79	42	90	151	50	167	163	331	251	0.32
Xu, 2016^55^	35	57	28	22	50	28	127	113	94	106	0.97
Taslim, 2017^56^	19	5	0	26	19	2	43	5	71	23	0.52
Rafik, 2019^57^	44	8	0	97	74	11	96	8	268	96	0.53

Abbreviation: HWE, Hardy-Weinberg equilibrium.

### Quality assessment

To evaluate the study quality, the control group of each study was tested for the HWE. One author (M.S) calculated the HWE for each study.

### Statistical analysis

One author (M.S) analyzed the data and other authors independently re-checked them; disagreements were resolved by discussion. The odds ratios (ORs) with the corresponding 95% confidence intervals (CIs) in all analyses were calculated by Review Manager 5.3 to evaluate the strength of the association between *MTHFR* C677T polymorphism and the risk of NSCL/P. To examine this association, we used five genetic models namely the allele (T vs. C), homozygote (TT vs. CC), heterozygote (CT vs. CC), dominant (TT + CT vs. CC), and recessive (TT vs. CC + CT) models. The Z test was used for evaluation of the significance of the pooled OR using both fixed effects (FE) (Mantel–Haenszel) and random effects (DerSimonian and Laird) models^19,20^. Heterogeneity across the studies was evaluated using both the Cochrane Q test^21,22^ and I^2^ metric^23,24^ ranging from 0 to 100%^25^. There was statistically significant heterogeneity if P-value < 0.1 and I^2^ > 50%; in that case, the random-effect model was used to estimate the pooled ORs and CI values. Otherwise, we used the fixed-effect model. The Chi-square test was used for calculation of the HWE for the control group of each study.

Subgroup analysis was performed according to the ethnicity and the source of cases to explore potential heterogeneity. The meta-regression analysis is a technique used to assess heterogeneity between the studies. This statistical approach determines whether there is a significant association between the study period and number of individuals with the pooled OR. The Begg’s funnel plot was carried out by the Comprehensive Meta-Analysis version 2.0 software identifying the standard error of log (OR), and the precision of each study was plotted against its log (OR)^25^. In addition, the results of Egger’s linear regression were retrieved using this software^26^. To estimate the consistency or stability of the results, we used sensitivity analysis namely cumulative analysis and one study was removed. P-value (2-tailed) <0.05 was statistically significant.

## Results

**Figure 1:**
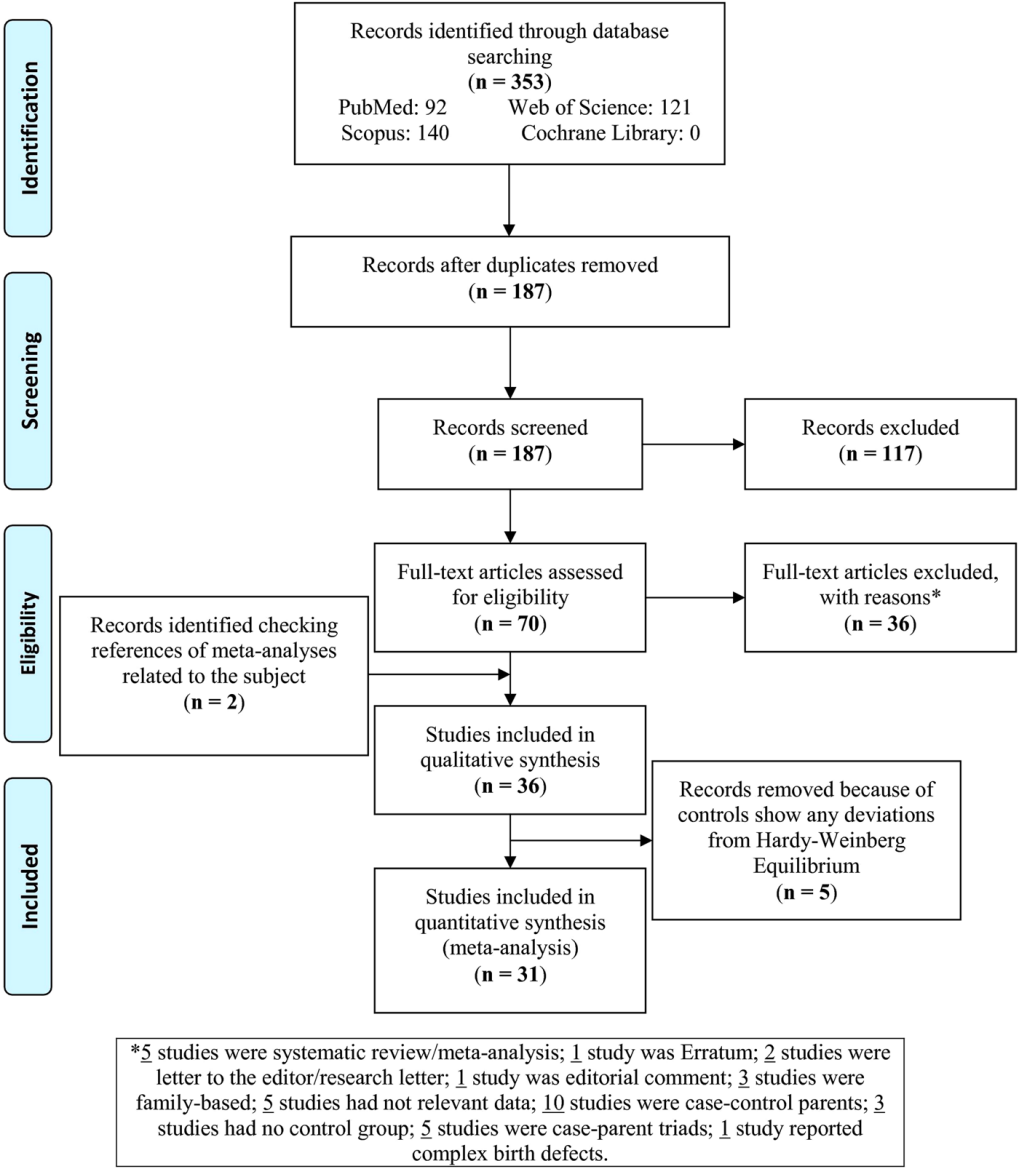
Flowchart of the study.

A total of 353 records were retrieved from the databases and after removing the duplicates, 187 records were screened (Fig. 1). Next, 117 records were excluded considering the eligibility criteria. Then, the full-texts of 70 articles were evaluated and 36 articles were excluded with reasons (five studies were systematic reviews/meta-analyses; one study was erratum; two studies were letter to editors/research letters; one study was editorial comment; three studies were family-based studies; five studies had irrelevant data; ten studies were case-control parents; three studies had no control group; five studies were case-parent triads; one study reported complex birth defects). On the other hand, by searching the references of meta-analyses, two other articles^27,28^ were found. Totally, 36 articles were included in this systematic review^6,27–61^ out of which, 5 studies^6,58–61^ had a deviation from the HWE for the control group and were excluded from the meta-analysis. Therefore, 31 articles were included and analyzed in this meta-analysis. In addition, one study^62^ was excluded for reducing bias compared with other previous meta-analyses, because it was a conference paper and therefore didn’t involve the eligibility criteria.

Table 1 shows the characteristics of each study included in this meta-analysis. The articles had been published from 1998 to 2019. Overall, the studies included 4,710 NSCL/P patients and 7,271 controls. Out of 31 studies, 10 studies were reported in mixed ethnicities, 10 studies had been conducted on Asians, and 11 studies had been conducted on Caucasians. In addition, the source of cases (patients) was population-based in 13 studies and hospital-based in 18 studies.

Table 2 shows the distribution of *MTHFR* C677T polymorphism genotype and allele in NSCL/P patients and controls. All studies followed the HWE for the control group.

### Meta-analysis

**Figure 2:**
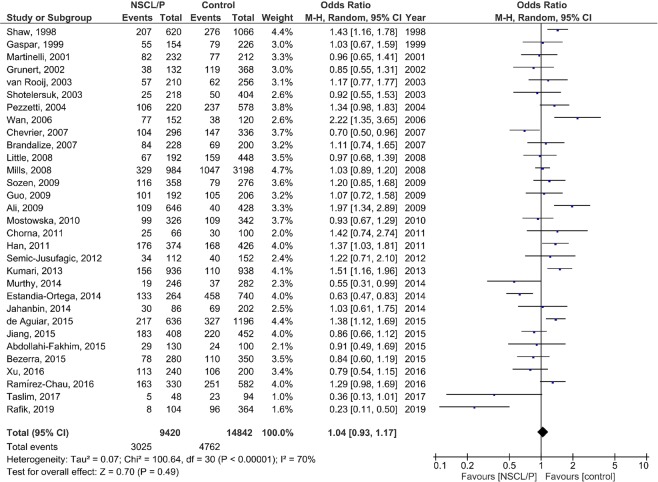
Random-effect forest plot of allele model (T vs. C) for the association between the NSCL/P risk and *MTHFR* C677T polymorphism.

**Figure 3:**
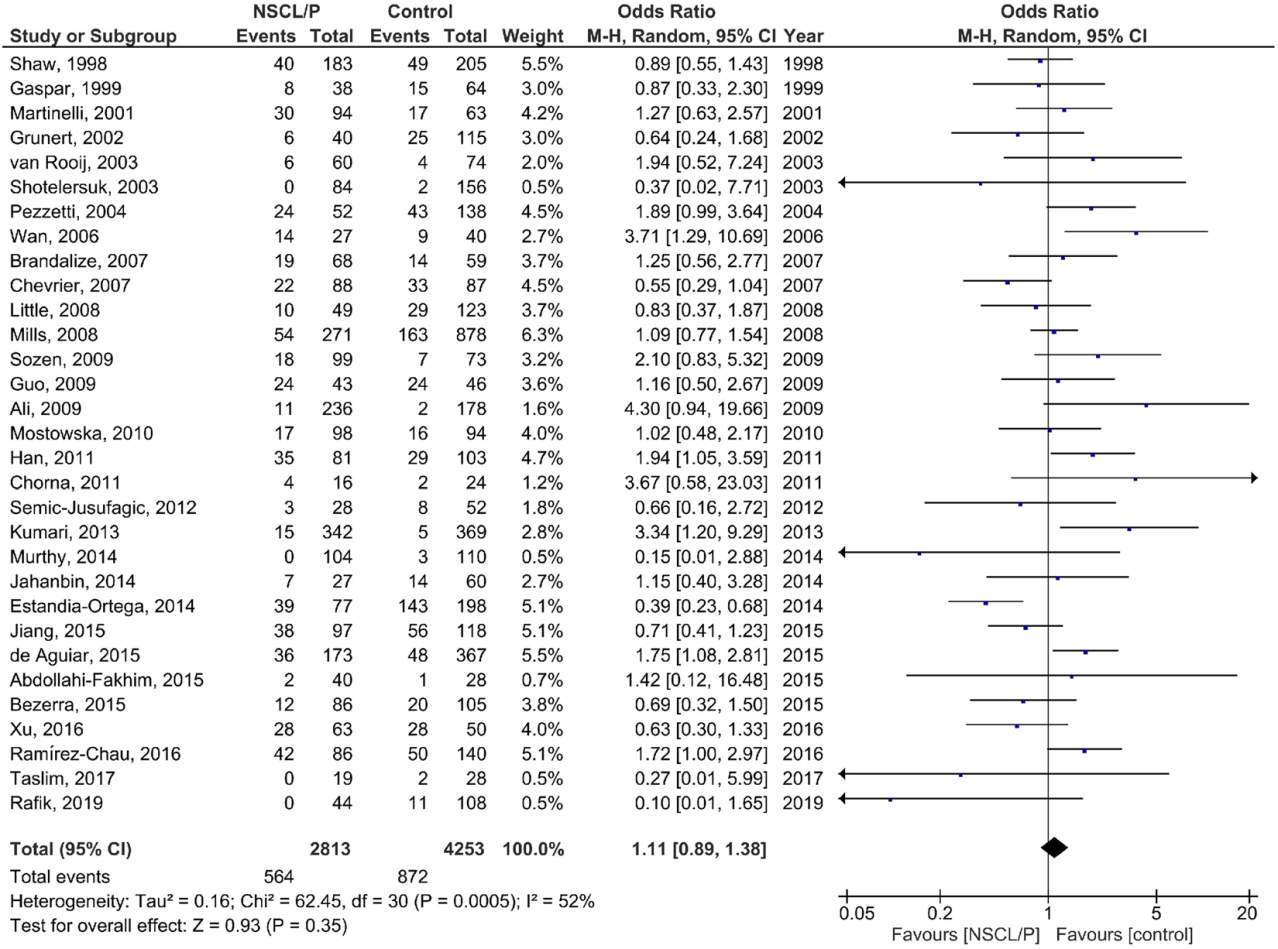
Random-effect forest plot of homozygote model (TT vs. CC) for the association between the NSCL/P risk and *MTHFR* C677T polymorphism.

**Figure 4:**
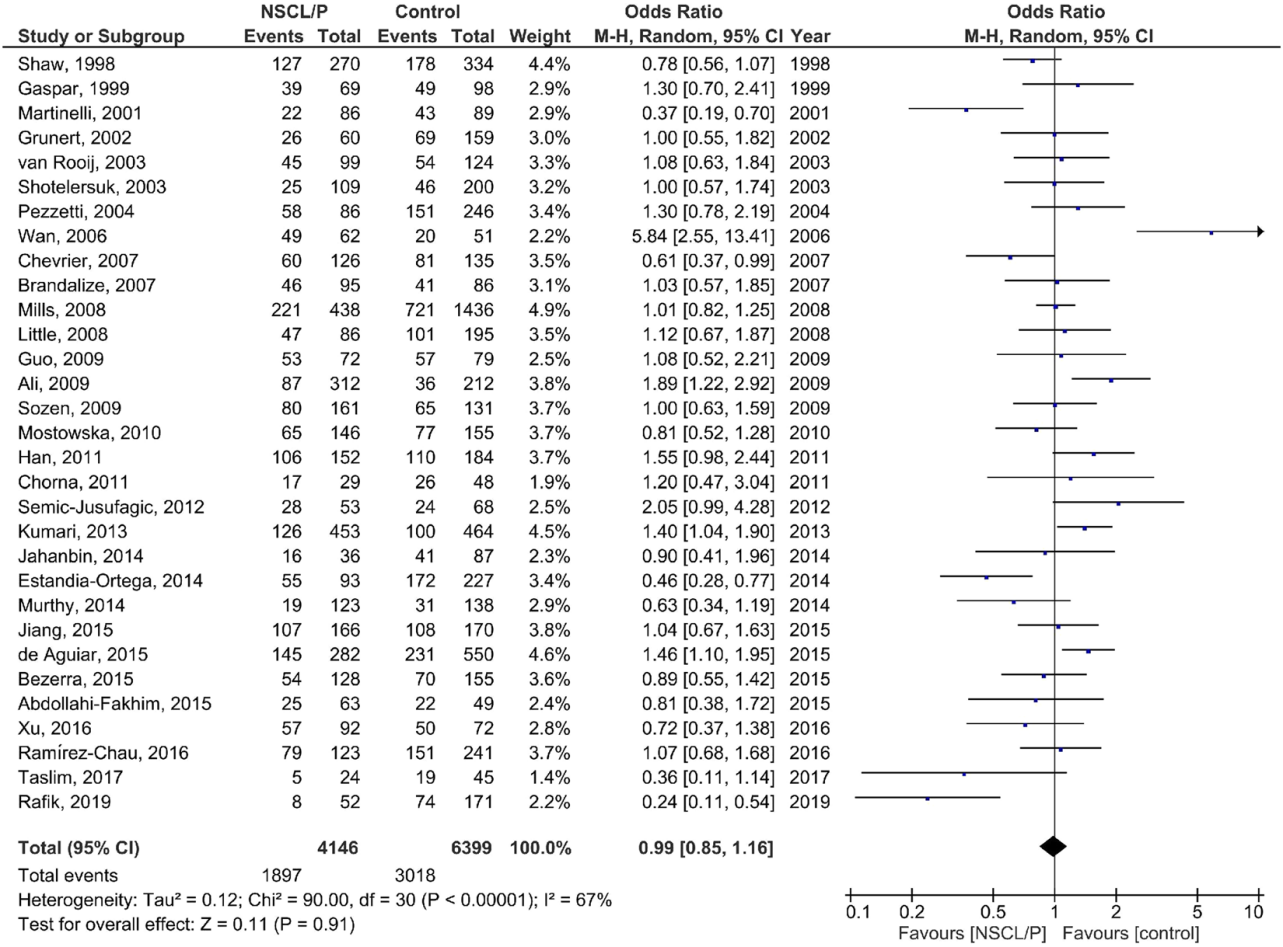
Random-effect forest plot of heterozygote model (CT vs. CC) for the association between the NSCL/P risk and *MTHFR* C677T polymorphism.

**Figure 5:**
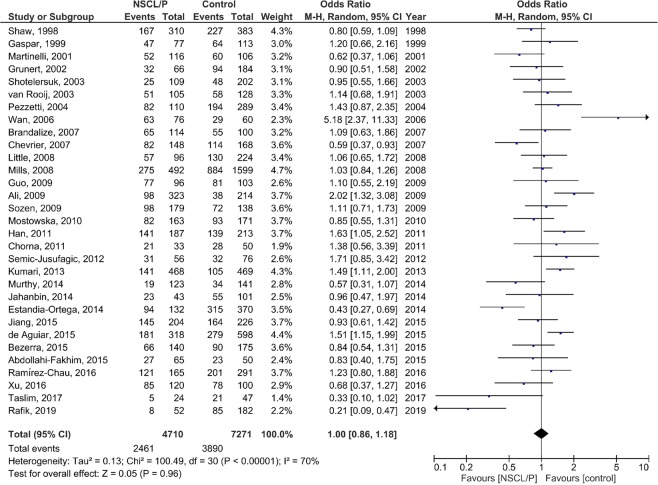
Random-effect forest plot of dominant model (TT + CT vs. CC) for the association between the NSCL/P risk and *MTHFR* C677T polymorphism.

**Figure 6:**
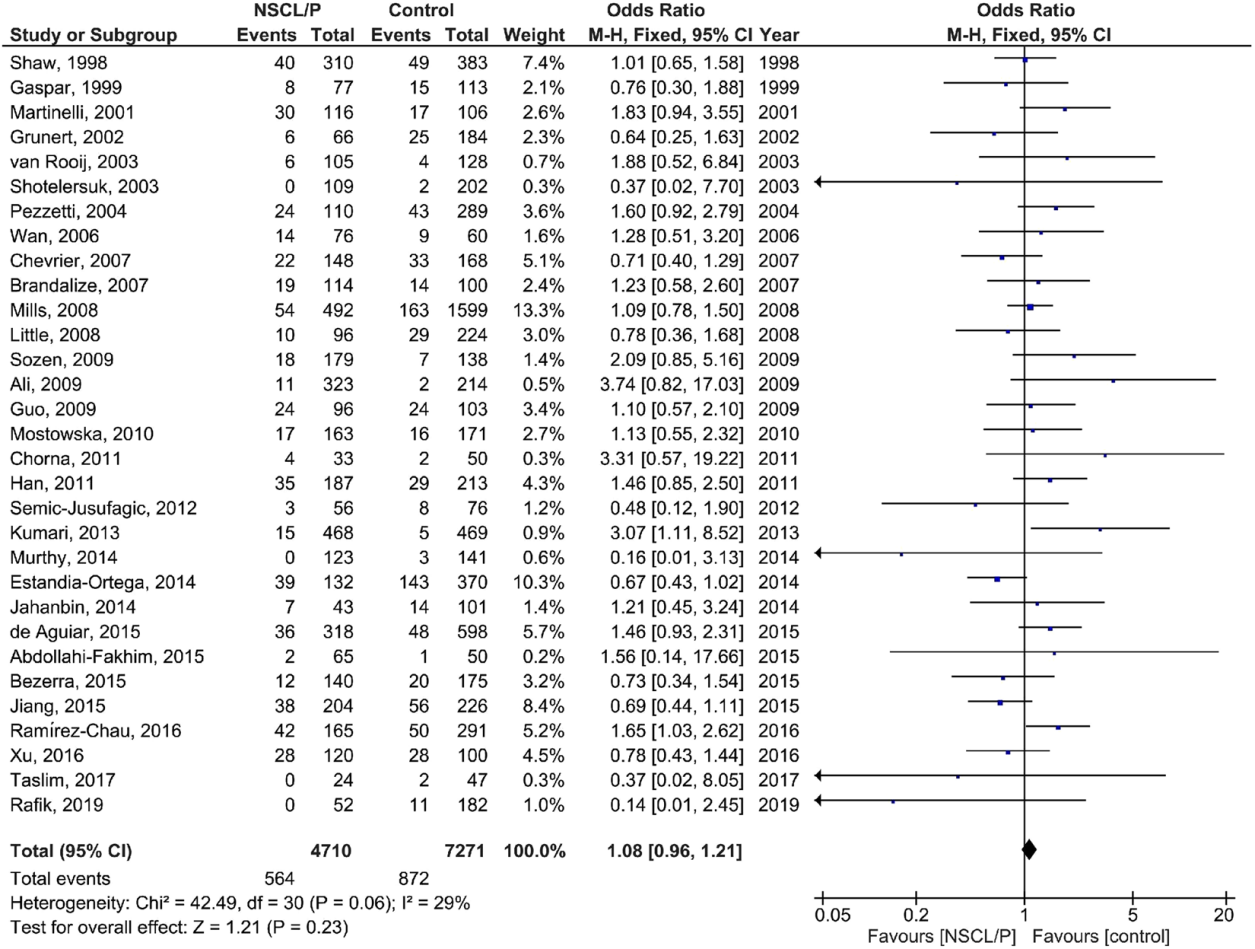
Random-effect forest plot of recessive model (TT vs. CC + CT) for the association between the NSCL/P risk and *MTHFR* C677T polymorphism.

The results of the pooled OR of the association between *MTHFR* C677T polymorphism and NSCL/P susceptibility are shown in Fig. 2 (T vs. C), Fig. 3 (TT vs. CC), Fig. 4 (CT vs. CC), Fig. 5 (TT + CT vs. CC), and Fig. 6 (T vs. CC + CT). Based on the results, there was no significant association between *MTHFR* C677T polymorphism and NSCL/P susceptibility related to allelic model [OR = 1.04; 95% CI: 0.93, 1.17; P = 0.49; I^2^ = 70% (P_heterogeneity_ or P_h_ < 0.00001)], homozygote model [OR = 1.11; 95% CI: 0.89, 1.38; P = 0.35; I^2^ = 52% (P_h_ = 0.0005)], heterozygote model [OR = 0.99; 95% CI: 0.85, 1.16; P = 0.91; I^2^ = 67% (P_h_ < 0.00001)], heterozygote model [OR = 0.99; 95% CI: 0.85, 1.16; P = 0.91; I^2^ = 67% (P_h_ < 0.00001)], dominant model [OR = 1.00; 95% CI: 0.86, 1.18; P = 0.96; I^2^ = 70% (P_h_ < 0.00001)], and recessive model [OR = 1.08; 95% CI: 0.96, 1.21; P = 0.23; I^2^ = 29% (P_h_ = 0.06)].

### Subgroup analysis

The subgroup analysis was performed based on the ethnicity and the source of cases for the association between *MTHFR* C677T polymorphism and NSCL/P risk (Table 3). There was no significant association between *MTHFR* C677T polymorphism and NSCL/P susceptibility with regard to the ethnicity (Asian, Caucasian, and mixed ethnicities) or the source of cases (population-based and hospital-based).

**Table 3:** Analysis of non-syndromic cleft lip/palate risk related to *MTHFR* C677T polymorphism according to ethnicity.

Study (n)	T vs. C	TT vs. CC	CT vs. CC	TT + CT vs. CC	TT vs. CC + CT
	OR (95% CI), I^2^ (%), P_h_	OR (95% CI), I^2^ (%), P_h_	OR (95% CI), I^2^ (%), P_h_	OR (95% CI), I^2^ (%), P_h_	OR (95% CI), I^2^ (%), P_h_
Overall (31)	1.04 (0.93, 1.17), 70, <0.00001	1.11 (0.89, 1.38), 52, 0.0005	0.99 (0.85, 1.16), 67, <0.00001	1.00 (0.86, 1.18), 70, <0.00001	1.08 (0.96, 1.21), 29, 0.06
**Ethnicity**					
Asian (10)	1.10 (0.85, 1.43), 77, <0.00001	1.34 (0.78, 2.29), 61, 0.006	1.21 (0.87, 1.67), 71, 0.0003	1.18 (0.84, 1.67), 76, <0.0001	1.06 (0.83, 1.35), 40, 0.09
Caucasian (11)	1.01 (0.92, 1.13), 12, 0.33	1.08 (0.86, 1.34), 13, 0.32	0.94 (0.82, 1.08), 47, 0.04	0.97 (0.85, 1.11), 29, 0.17	1.13 (0.92, 1.39), 8, 0.37
Mixed (10)	0.99 (0.79, 1.24), 81, <0.00001	0.99 (0.67, 1.46), 67, 0.001	0.89 (0.68, 1.17), 71, 0.0003	0.89 (0.66, 1.19), 77, <0.00001	1.04 (0.87, 1.26), 45, 0.06
**Source of cases**					
PB (13)	1.10 (0.94, 1.28), 62, 0.002	1.01 (0.81, 1.25), 29, 0.16	1.00 (0.81, 1.24), 61, 0.002	1.03 (0.85, 1.24), 57, 0.006	1.04 (0.86, 1.27), 37, 0.09
HB (18)	1.00 (0.84, 1.18), 75, <0.00001	1.11 (0.81, 1.53), 60, 0.0005	0.98 (0.78, 1.24), 71, <0.00001	0.98 (0.77, 1.25), 77, <0.00001	1.10 (0.94, 1.28), 26, 0.15

Abbreviations: PB, population-based; HB, hospital-based. *P-values were insignificant (P > 0.05) in all analyses. **P_h_ means P_heterogeneity_.

### Meta-regression

**Figure 7:**
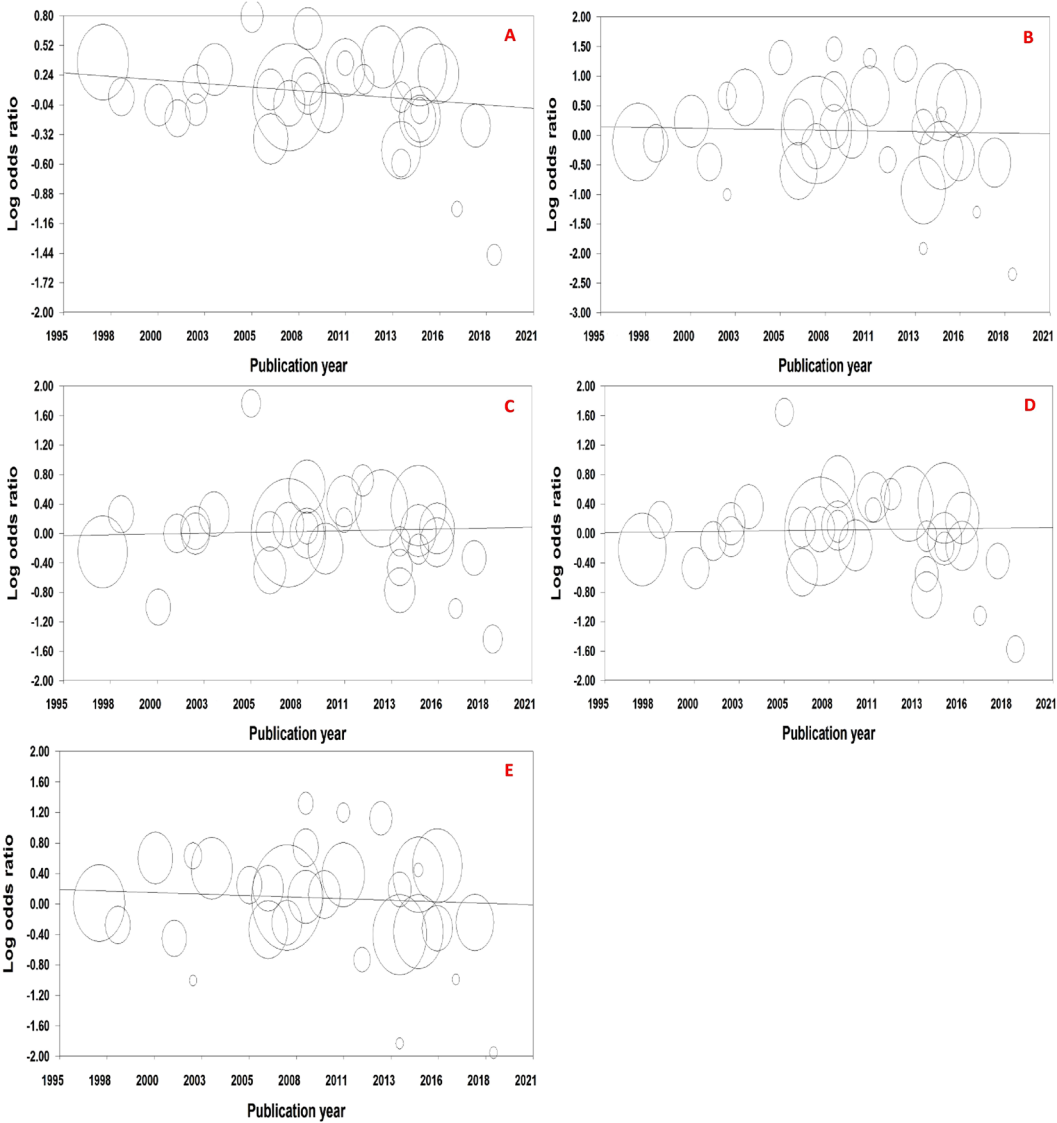
Fixed-effect meta-regression of log odds ratio versus publication year for (**A**) allele model, (**B**) homozygote model, (**C**) heterozygote model, (**D**) dominant model, and (**E**) recessive model.

**Figure 8:**
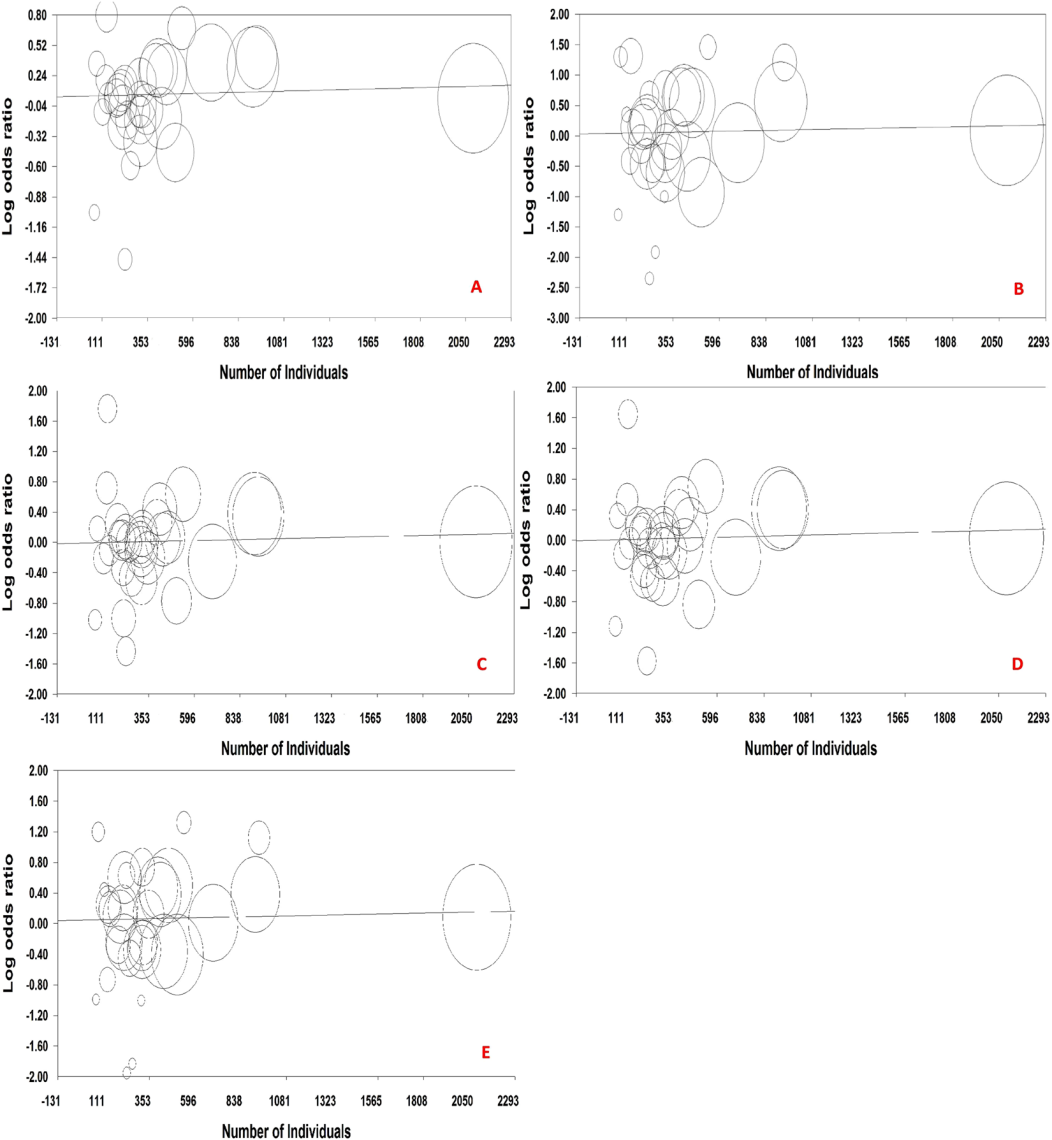
Fixed-effect meta-regression of log odds ratio versus number of individuals for (**A**) allele model, (**B**) homozygote model, (**C**) heterozygote model, (**D**) dominant model, and (**E**) recessive model.

Considering the year of publication and the number of individuals as independent variables and the log (OR) as the dependent variable, the fixed-effect meta-regression results are presented in Table 4, Figs. 7 and 8. To estimate the functional relationship of the log OR with the year of publication and the number of individuals, the analysis showed only a significant relationship for the allele model (T vs. C) for the year of publication with a regression coefficient of −0.01346. Therefore, there was a significant linear relationship between the year of publication and log ORs for the allele model (T vs. C), but not for the genetic models.

**Table 4:** Fixed-effect meta-regression of log odds ratio for the publication year and number of individuals.

Models for year of publication		Point Estimate	Standard Error	Lower Limit	Upper Limit	Z-value	P
T vs. C	Slope	−0.01346	0.00548	−0.02420	−0.00271	−2.45454	**0.01411**
	Intercept	27.12064	11.01569	5.53028	48.71099	2.46200	0.01382
TT vs. CC	Slope	−0.00466	0.01212	−0.02842	0.01910	−0.38437	0.70071
	Intercept	9.44414	27.35460	−38.29000	57.17828	0.38778	0.69818
CT vs. CC	Slope	0.00449	0.00801	−0.01122	0.02019	0.55990	0.57555
	Intercept	−8.98145	16.09724	−40.53146	22.56857	−0.55795	0.57688
TT + CT vs. CC	Slope	0.00449	0.00801	−0.01122	0.02019	0.55990	0.57555
	Intercept	−8.98145	16.09724	−40.53146	22.56857	−0.55795	0.57688
TT vs. CC + CT	Slope	−0.00788	0.01100	−0.02945	0.01369	−0.71614	0.47391
	Intercept	15.91868	22.11551	−27.42.692	59.26427	0.71980	0.47165
**Models for number of individuals**		**Point Estimate**	**Standard Error**	**Lower Limit**	**Upper Limit**	**Z-value**	**P**
T vs. C	Slope	0.00004	0.00005	−0.00005	0.00014	0.89668	0.36989
	Intercept	0.05212	0.04523	−0.03652	0.14076	1.15249	0.24912
TT vs. CC	Slope	0.00006	0.00011	−0.00015	0.00027	0.56260	0.57371
	Intercept	0.04092	0.10171	−0.15842	0.24026	0.40233	0.68744
CT vs. CC	Slope	0.00006	0.00007	−0.00008	0.00019	0.83343	0.40460
	Intercept	−0.00968	0.06556	−0.13818	0.11882	−0.14765	0.88262
TT + CT vs. CC	Slope	0.00006	0.00006	−0.00006	0.00019	0.97564	0.32924
	Intercept	0.00136	0.06221	−0.12057	0.12329	0.02180	0.98261
TT vs. CC + CT	Slope	0.00005	0.00010	−0.00015	0.00025	0.48796	0.62558
	Intercept	0.04821	0.09159	−0.13129	0.22771	0.52639	0.59862

### Publication bias

**Figure 9:**
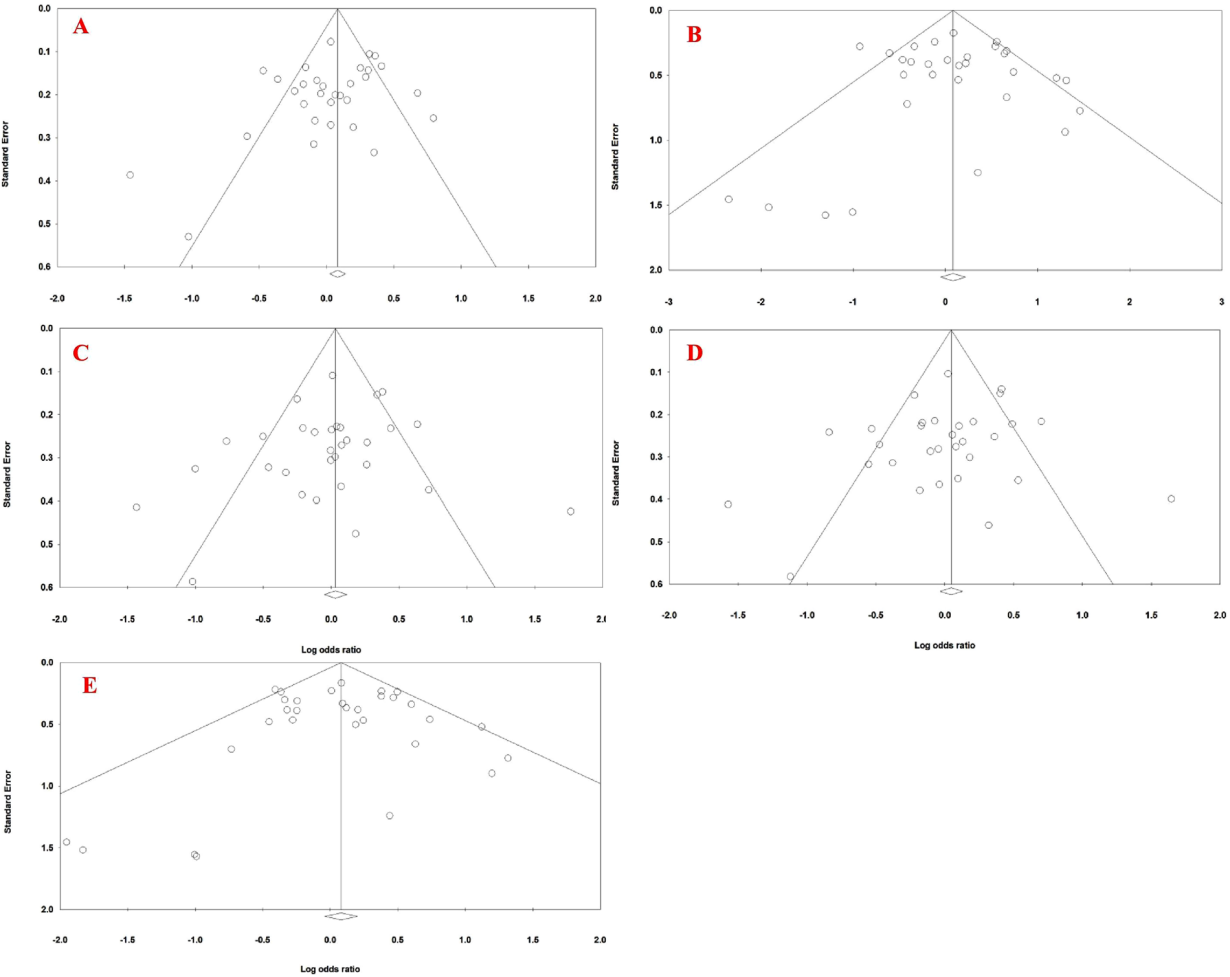
Funnel plot of (**A**) allele model, (**B**) homozygote model, (**C**) heterozygote model, (**D**) dominant model, and (**E**) recessive model for the association between the NSCL/P risk and *MTHFR* C677T polymorphism (fixed-effects model).

Figure 9 shows the funnel plots of all genetic models to evaluate the association between the NSCL/P risk and *MTHFR* C677T polymorphism in a fixed-effect model. There was no publication bias between the NSCL/P risk and *MTHFR* C677T polymorphism in the genetic models. The P-values of Begg’s/Egger’s tests were 0.21470/0.12123, 0.95933/0.97596, 0.22753/0.29895, 0.25480/0.28137, and 0.93228/0.91342 for T vs. C, TT vs. CC, CT vs. CC, TT + CT vs. CC, and TT vs. CC + CT, respectively.

### Sensitivity analysis

Two analyses (one study excluded and cumulative analysis) were performed and the pooled ORs did not change qualitatively. Therefore, the analyses showed that the pooled ORs under all genetic models were stable and trustworthy.

## Discussion

NSCL/P is one of the most common congenital anomalies with high rate of mortality. Its pathogenesis is difficult to be attributed to either environmental or genetic factors. The pathway of folate metabolism plays a significant role in the synthesis, repair, and methylation of DNA involved in NSCL/P pathogenesis^63^. MTHFR enzyme plays an important role in folate intake, and mutations of *MTHFR* gene significantly impact the stability and thus the function of the enzyme; *MTHFR* C677T is the most common mutation of this gene^64^. *MTHFR* C677T polymorphism is related to a reduction in MTHFR activity, raised plasma homocysteine concentration, and lower plasma level of folic acid, which consequently contribute to NSCL/P^65^. The present meta-analysis was performed to more precisely assess the relationship between *MTHFR* C677T polymorphism and NSCL/P susceptibility. In pooled analysis, the meta-analysis showed no significant association between *MTHFR* C677T polymorphism and NSCL/P risk.

Out of 31 studies included in the present meta-analysis, six studies^27,34,39,44,45,52^, five studies^34,39,44,46,52^, and four studies^34,39,46,52^ reported significantly increased risk of T allele, TT genotype, and CT genotype in NSCL/P patients compared with controls, respectively. Also, five studies^36,47,49,55,57^, two studies^47,57^, and four studies^28,36,47,57^ reported a significantly decreased risk of T allele, TT genotype and CT genotype in NSCL/P patients compared with controls, respectively. In addition, TT + CT genotype was reported to have a significantly increased risk in five studies^34,39,44,46,52^ and significantly decreased risk in four studies^36,47,55,57^ in NSCL/P patients compared with controls. Based on the recessive model, three studies^39,46,54^ reported significantly increased risk of TT genotype and one study^57^ reported its significantly decreased risk in NSCL/P patients compared with controls.

A recent meta-analysis with 24 case-control studies^14^ investigating the relationship between NSCL/P and *MTHFR* C677T polymorphism showed that the TT genotype was a risk factor for NSCL/P in Asians in homozygote (OR = 1.96, *P* < 0.001) and recessive (OR = 1.45, *P* = 0.028) models. Also, based on mothers with NSCL/P progeny versus control mothers with healthy progeny in 10 studies, the TT genotype of Caucasian mothers may increase progeny NSCL/P morbidity. Another recent meta-analysis of 22 case-control studies^15^ showed that *MTHFR* C677T polymorphism was associated with a higher risk of NSCL/P. Both meta-analyses also included studies with a deviation of HWE. However, in the present meta-analysis, we excluded such studies from the pooled analysis and therefore, reviewed 31 case-control studies and had lower heterogeneity compared with the meta-analysis with 22 studies^15^. Almost similar to the findings of a meta-analysis with 24 studies^14^, our results showed no association between *MTHFR* C677T and susceptibility to NSCL/P. In one meta-analysis^15^, definition of ethnicity was different from that in another study^14^ and our meta-analysis. The meta-regression showed a linear relationship with a negative slope between the year of publication and log ORs for the allele model and therefore by increasing years of publication, the risk of T allele decreased in NSCL/P patients compared with controls. There were two other meta-analyses with eight^16^ and nine^7^ case-control studies related to our topic. One of them^16^ reported no association and another one on Asian ethnicity showed a significant association between *MTHFR* C677T and susceptibility to NSCL/P. Luo *et al*.^17^ on nine studies in a meta-analysis didn’t show any evidence for significant association between infant or maternal *MTHFR* C677T polymorphism and NSCL/P risk, but suggested that maternal *MTHFR* 677TT polymorphism could increase the risk of having a NSCL/P offspring in the white population. Pan *et al*.^8^ on seventeen studies showed that this polymorphism was a risk factor involved in the development of NSCL/P in Asians that definition of ethnicities in this meta-analysis was different from our meta-analysis. The results showed that the effect of each factor alone on the association was low, but such a high heterogeneity among the studies could be due to simultaneous effect of several factors such as differences in the ethnicity of the study populations, source of cases, and number of individuals.

This study had several important limitations including high heterogeneity across studies, unadjusted ORs used in the studies, and intake of folic acid and other supplements that were not considered. Nevertheless, the present study included more studies with meta-regression without any deviation of HWE for controls in all studies compared with other meta-analyses. It did not have publication bias, and the results were stable.

In conclusion, the result of the present meta-analysis revealed that *MTHFR* C677T polymorphism is not associated with susceptibility to NSCL/P, and the subgroup analyses based on the ethnicity and the source of cases further confirmed this result. However, well-designed studies with larger sample size are required taking into account the role of micronutrients such as folic acid in NSCL/P risk.
